# Comparative Genomic Analysis Across Multiple Species to Identify Candidate Genes Associated with Important Traits in Chickens

**DOI:** 10.3390/genes16060627

**Published:** 2025-05-24

**Authors:** Fuyang Zhang, Hengcong Chen, Cheng Chang, Jiamei Zhou, Hui Zhang

**Affiliations:** 1 Key Laboratory of Chicken Genetics and Breeding, Ministry of Agriculture and Rural Affairs, Harbin 150030, China; s220501010@neau.edu.cn (F.Z.); 15610303963@163.com (H.C.); changcheng20200911@163.com (C.C.); zhoujiamei@neau.edu.cn (J.Z.); 2 Key Laboratory of Animal Genetics, Breeding and Reproduction, Education Department of Heilongjiang Province, Harbin 150030, China; 3 College of Animal Science and Technology, Northeast Agricultural University, Harbin 150030, China

**Keywords:** chicken, comparative genomics, gene families, candidate genes, functional annotation

## Abstract

**Background:** As one of the most important poultry species worldwide, chickens provide substantial amounts of meat, eggs, and other products for human consumption. With continuous improvements in living standards, consumer demand for high-quality animal products is increasing, making it essential to understand the genetic basis of key traits such as egg production, meat quality, and disease resistance for targeted genetic improvement. **Methods:** In this study, a number of the candidate genes associated with important traits in chickens were screened by various comparative genomics analysis methods. To further clarify the relationship between these candidate genes and important traits in chickens, they were functionally annotated through the KOG, GO, and KEGG databases. **Results:** These candidate genes are mainly concentrated in the functional categories of transcription and signal transduction mechanisms and are involved in biological processes such as cyclic nucleotide biosynthesis and intracellular signaling, which involve signaling pathways such as ECM–receptor interactions and calcium signaling. **Conclusions:** Based on the annotation results from various databases, a functional search of the candidate genes and related literature reports, the following results were obtained: genes such as *TBX22*, *LCORL*, and *GH* were associated with chicken growth traits; genes such as *A-FABP*, *H-FABP*, and *PRKAB2* were associated with chicken meat quality; genes such as *IGF-1*, *SLC25A29*, and *WDR25* were associated with chicken reproductive traits; and genes such as *C1QBP*, *VAV2* and *IL12B* were associated with chicken disease resistance traits. Overall, the findings of this study provide novel insights and candidate genes for genetic improvements in chickens, laying a foundation for future research and breeding strategies targeting key economic traits.

## 1. Introduction

Chicken (*Gallus gallus*) plays a vital role in the global agricultural economy as one of the most important poultry species worldwide [1]. With the continuous growth of the global population and the improvement in living standards, the demand for animal-based food products is steadily increasing [2]. Among them, chicken meat and eggs account for a growing share of global food consumption each year [3]. As a result, improving key production traits in chickens such as egg-laying performance, growth rate, meat quality, and disease resistance, has become a central focus in the development of both the broiler and layer industries [4]. Compared with nutritional or environmental interventions, genetic approaches offer a more effective means of enhancing chicken production traits, particularly complex economic traits and polygenic characteristics [5]. Traditional breeding methods rely primarily on phenotypic selection, which limits breeding efficiency, especially in the improvement of polygenic traits—an ongoing challenge in poultry breeding programs [6].

In recent years, the rapid advancement of genomics and high-throughput sequencing technologies have positioned genomic approaches as powerful tools in genetic breeding research [7]. As a sub-discipline of genomics, comparative genomics has been widely utilized in the investigation of gene function and species evolution [8]. By analyzing similarities and differences among the genomes of various species, researchers can identify conserved genes, expanded gene families, and genes that may have undergone positive selection [9]. These genomic features are often closely linked to biological characteristics, key traits, and adaptive evolution [10]. Through cross-species genomic comparisons, the unique evolutionary roles of specific genes in chickens can be uncovered, aiding in the identification of the candidate genes associated with economically important traits [11]. Such comparisons also help reveal gene-specific selective pressures experienced during chicken evolution, many of which are likely involved in species adaptation and trait development, thereby offering potential targets for molecular breeding [12]. For instance, He et al. [13] investigated the genomes of cultivated and wild buckwheat, revealing that increases in the copy numbers of the *FdCHI, FdF3H*, *FdDFR*, and *FdLAR* gene families were associated with elevated levels of medicinal flavonoids. A resequencing of 34 wild buckwheat ecotypes further identified genes such as *FdMYB44* and *FdCRF4*, which may be involved in flavonoid biosynthesis and plant structural differentiation, respectively. Liu et al. [14] performed comparative genomic analyses between a giraffe and other ruminants, identifying *FGFRL1* as a potential key gene contributing to the giraffe’s remarkable height. Similarly, Kohl et al. [15] compared poultry genomes with those of other birds, revealing genome rearrangement and duplication events during avian evolution. Their work contributed to the reconstruction of a phylogenetic tree, shedding light on avian ancestry and evolutionary pathways.

Comparative genomics research encompasses a wide range of analytical methods used to investigate genomic similarities and differences among species [16]. These approaches span various aspects, including gene family classification and functional analysis, genomic structural variation, estimations of species divergence times, and phylogenetic tree construction [17]. The commonly employed methods include a gene family clustering analysis, divergence time estimations, phylogenetic reconstruction, a gene family expansion and contraction analysis, a positive selection analysis, a genomic collinearity (synteny) analysis, whole-genome duplication (WGD) detection, and an insertion time analysis of long terminal repeat retrotransposons (LTR-RT) [18]. These methodologies collectively enable researchers to explore the evolution of gene families, infer evolutionary relationships among species, detect selective pressures on genes, and assess both conservation and variability in genome structure [19]. Together, they provide valuable insights into genetic diversity, evolutionary history, and species-specific adaptive changes [20].

This study aimed to identify the candidate genes associated with important traits in chicken through comparative genomic analysis between chickens and other species. By examining differences in genome structures across species, and subsequently conducting functional annotations, we sought to elucidate the relationships between these candidate genes and key phenotypic traits. The goal was to provide novel insights and potential genetic targets for chicken breeding, thereby laying a foundation for the genetic improvement of economically important traits in chicken.

## 2. Materials and Methods

### 2.1. Genome Data Acquisition

The genome assemblies of a chicken (*G. gallus*), duck (*Anas platyrhynchos*), goose (*Anser cygnoides*), cow (*Bos taurus*), sheep (*Ovis aries*), pig (*Sus scrofa*), human (*Homo sapiens*), and zebrafish (*Danio rerio*) were obtained from the NCBI Genome Database (https://www.ncbi.nlm.nih.gov/data-hub/genome/) and Ensembl (https://www.ensembl.org/index.html?redirect=no), accessed on 16 March 2024. For each species, the reference genome assembly, protein sequences, and annotation files in GFF format were downloaded. All datasets used in this study are based on the most recent reference genome versions available at the time of analysis. A summary of species-specific information is provided in Supplementary Table S1, and the corresponding genome download links are listed in Supplementary Table S2.

### 2.2. Gene Family Clustering Analysis

Protein sequences corresponding to the longest transcripts of protein-coding genes from eight species—chickens, ducks, geese, cows, sheep, pigs, humans, and zebrafish—were extracted for gene family clustering. Orthologous gene families were identified using OrthoFinder (v2.4.0) [21], with sequence similarity searches performed using DIAMOND and an E-value threshold of 0.001.

### 2.3. Phylogenetic Tree Construction and Differentiation Time Calculation

The protein sequences of single-copy orthologous genes identified using OrthoFinder were used for phylogenetic tree construction. Multiple sequence alignments were performed using MAFFT (v7.205) [22] with the parameters --localpair and --maxiterate 1000. Poorly aligned or highly divergent regions were removed using Gblocks (v0.91b) [23] with the parameter −b5 = h. The filtered alignments of all gene families were then concatenated into a supergene sequence for each species. Model selection was performed using Model Finder, integrated within the IQ-TREE software (v2.2.0) [24], and the best-fit model identified was JTT+F+I+G4. A maximum likelihood (ML) phylogenetic tree was subsequently constructed using this model, with 1000 bootstrap replicates to assess node support.

### 2.4. Analysis of Gene Family Expansion and Contraction

Based on the identified complete gene families and the species phylogenetic tree with estimated divergence times, gene family expansion and contraction analyses were performed using CAFE software (v4.2) [25]. Conditional *p*-values were calculated for each gene family, and families with a *p*-value less than 0.05 were considered to have undergone significant expansion or contraction. The number of gene families exhibiting significant expansion or contraction during the evolutionary process of chickens was then quantified. Subsequently, a hypergeometric test algorithm was used to calculate the Q-value (FDR, i.e., false discovery rate) to exclude false-positive results by using the R package (https://github.com/StoreyLab/qvalue), accessed on 30 April 2024.

### 2.5. Positive Choice Analysis

A positive selection analysis was conducted using the CodeML module of the PAML [26] software (v4.9i) package. Single-copy orthologous gene families from chickens, ducks, geese, cows, sheep, pigs, humans, and zebrafish were used in the analysis. The protein sequences of each gene family were aligned using MAFFT with the parameters --localpair and --maxiterate 1000, and codon alignments were generated using PAL2NAL by converting the protein alignments back into nucleotide sequences. Subsequently, the branch-site model in CodeML was applied to detect positively selected genes, using the F3x4 model of codon frequency. Two models were compared: Model A (which allows for positive selection on specific sites in the foreground branch, ω > 1) and the null model (which does not allow any sites with ω > 1). A likelihood ratio test (LRT) was performed using the “chi2” program in PAML to determine whether the difference between the models was statistically significant (*p* < 0.05). The Bayes Empirical Bayes (BEB) method was then used to estimate the posterior probabilities of specific sites being under positive selection, with sites showing a posterior probability > 0.95 considered to be under significant positive selection. The genes containing such sites were identified as candidate genes under positive selection.

### 2.6. Genome-Wide Replication Event Analysis

Whole-genome duplication (WGD) refers to the multiplication of the entire genome content. In this study, WGD events between chickens and other species were identified using the synonymous substitution rate (Ks) method, implemented via the WGD software (v3.7.3) [27]. The timing of WGD events was estimated using the formula Ks/2r, where represents the neutral substitution rate.

### 2.7. Genomic Covariance Analysis

A collinearity (synteny) analysis was performed by identifying homologous gene pairs between species using DIAMOND (v0.9.29.130) [28] with an E-value threshold of 1e-5 and a C-score cutoff of > 0.5. The C-score filtering was conducted using JCVI (v0.9.13) [29]. Homologous gene pairs were then assessed for chromosomal proximity based on GFF3 annotation files, and syntenic blocks were identified using MCScanX [30] (parameter: −m 100). All genes within the collinear blocks were retrieved for further analysis. Finally, synteny plots for each species pair were generated using JCVI.

### 2.8. LTR-RT Insertion Time Analysis

Long terminal repeat retrotransposons (LTRs) were identified using LTR-harvest (v1.5.10) [31] and LTR-Finder (v1.07) [32]. Redundant LTRs were filtered out using LTR-retriever [33]. Flanking sequences on either side of the LTRs were extracted and aligned using MAFFT (v7.205). The insertion times of LTRs were then estimated using the Kimura two-parameter model implemented in EMBOSS (v6.6.0) [34].

### 2.9. Annotation and Pathway Analysis

The identified genes in the chicken-specific gene families, the genes in gene families that underwent significant expansion during evolution, and the genes subject to positive selection pressure were compared with the KOG, KEGG, and GO databases for annotation and pathway analysis. KOG annotations were obtained using the online tool eggNOG-mapper (v2.1.9; http://eggnog-mapper.embl.de/), accessed on 2 June 2024, and visualized using the COG function on the OmicShare platform (https://www.omicshare.com/tools/Home/Soft/cog), accessed on 2 June 2024. A Gene Ontology (GO) enrichment analysis was performed using the R package cluster Profiler (v4.0), with annotations from the org.Ss.eg.db database (v3.16.0), and results were visualized using the built-in dotplot function. KEGG pathway enrichment was conducted by accessing the KEGG database (https://www.kegg.jp/), accessed on 4 June 2024, through the KEGG API via the cluster Profiler (v4.0), and the results were also visualized using the dotplot function.

## 3. Results

### 3.1. Identification of Candidate Genes Associated with Key Chicken Traits via Gene Family Clustering Analysis

#### 3.1.1. Distribution of Gene Family Copy Number by Species

**Figure 1:**
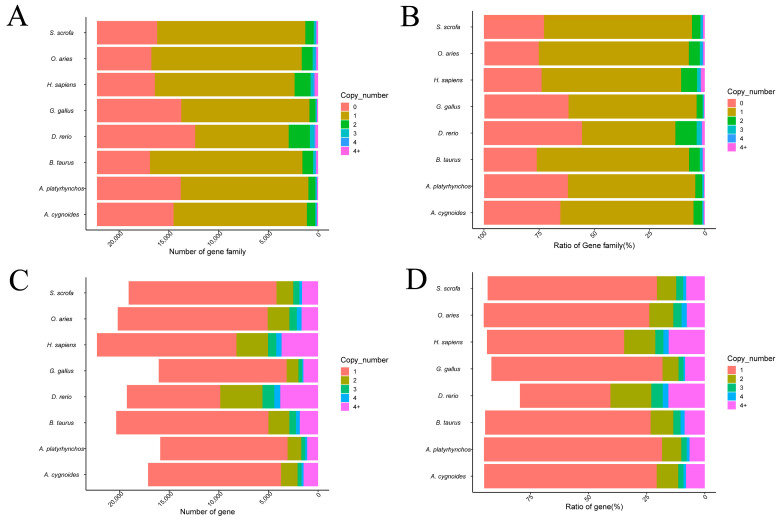
Distribution of gene families and gene copy numbers across eight species. (**A**,**B**) Distribution of gene copy numbers within all gene families across species. (**C**,**D**) Distribution of gene copy numbers at the gene level across species. Gene families and genes were categorized based on copy numbers (0, 1, 2, 3, 4, and >4), and the proportion in each category was calculated for each species.

To gain a comprehensive understanding of the distribution of gene families and gene copy numbers across different species, and to provide foundational data for subsequent comparative genomic analyses, this study quantified the number of gene copies within each gene family across eight species: chickens, ducks, geese, cows, sheep, pigs, humans, and zebrafish. The analysis included statistics on the number and proportion of gene families and genes with 0, 1, 2, 3, 4, and more than 4 copies in each species. These data reflect both the presence and expansion of gene families, offering insights into species-specific gene duplication events. The detailed statistical results are presented as follows:

The copy number distribution analysis of gene families revealed that the majority of gene families in each species contained a single gene copy. Moreover, the overall pattern of gene family copy number distribution was largely consistent across the species analyzed (Figure 1A,B). Similarly, the distribution of individual gene copy numbers within each species showed that genes with a single copy accounted for the majority of all genes. This pattern was also found to be broadly conserved among the different species (Figure 1C,D).

#### 3.1.2. Identification and Statistics of Species-Specific Gene Families

**Figure 2:**
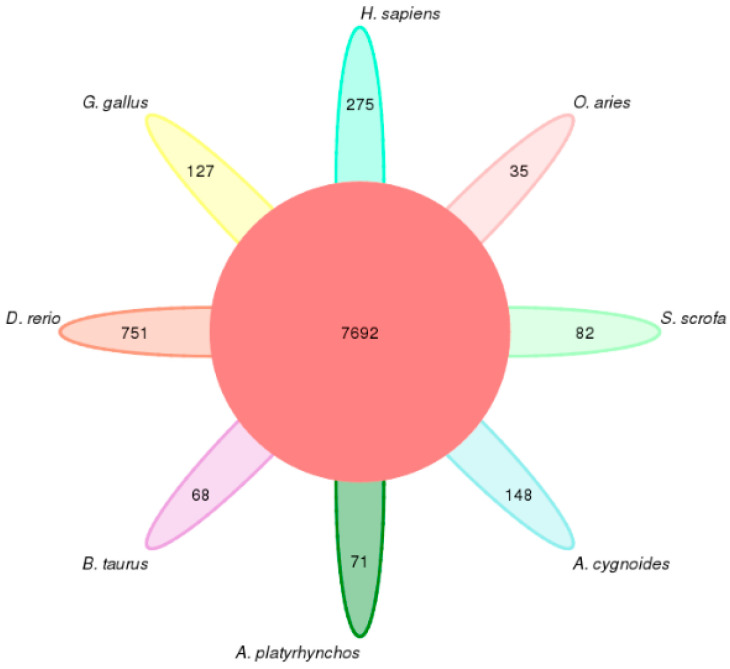
Petal diagram of gene family clustering among eight species. The petal map illustrates the distribution of shared and species-specific gene families across the eight analyzed species: chickens, ducks, geese, cows, sheep, pigs, humans, and zebrafish. The central region represents the number of gene families common to all species, while each petal indicates the number of gene families unique to a specific species.

The numbers of shared and species-specific gene families among chickens, ducks, geese, cows, sheep, pigs, humans, and zebrafish were analyzed. As shown in Figure 2, a total of 7692 gene families were common to all eight species. Additionally, 127 gene families were found to be specific to chickens, 71 to ducks, and 82 to pigs.

#### 3.1.3. Statistics on the Number of Gene Families Specific to Each Species

A cluster analysis of protein sequences from eight species—chickens, ducks, geese, cows, sheep, pigs, humans, and zebrafish—revealed that the chicken genome contains 17,489 genes. Among these, 16,050 genes (91.80%) were clustered into 13,742 gene families, while 1439 genes remained uncluttered. A total of 127 gene families were identified as chicken-specific, comprising 824 genes (Table 1).

### 3.2. Screening Candidate Genes Related to Important Chicken Traits Based on Gene Family Expansion and Contraction Analysis

**Figure 3:**
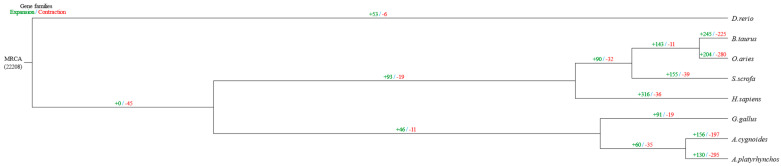
Phylogenetic trees show gene family expansion and contraction events across species. The evolutionary tree illustrates the relationships among the eight species analyzed, along with the number of gene families that have undergone significant expansion or contraction. Green numbers indicate the number of expanded gene families, while red numbers indicate the number of contracted gene families along each lineage.

The results of the contraction and expansion of chicken gene families indicated that 91 gene families underwent significant expansion and 19 gene families underwent significant contraction during the evolutionary process of chickens, and the genes in the gene families that underwent significant expansion may have played an important role in the formation of specific traits during the evolutionary process of chickens. The relationship between these genes and traits was further clarified through functional annotation. The specific results and gene family expansion and contraction in other species are shown in Figure 3.

### 3.3. Screening Candidate Genes Related to Important Chicken Traits Based on Positive Selection Analysis

The results of the positive selection analysis showed that 27 genes, including *GTSF1*, *GRAP2*, *RCN1L*, and *AGBL4*, were subjected to positive selection pressure (Table 2), and these genes may have played important roles in the formation of specific traits during the evolution of chickens. In order to further unravel the relationship between these genes and the traits, their potential biological functions and regulatory mechanisms were investigated.

### 3.4. Phylogenetic Tree Construction and Differentiation Time Calculation

**Figure 4:**
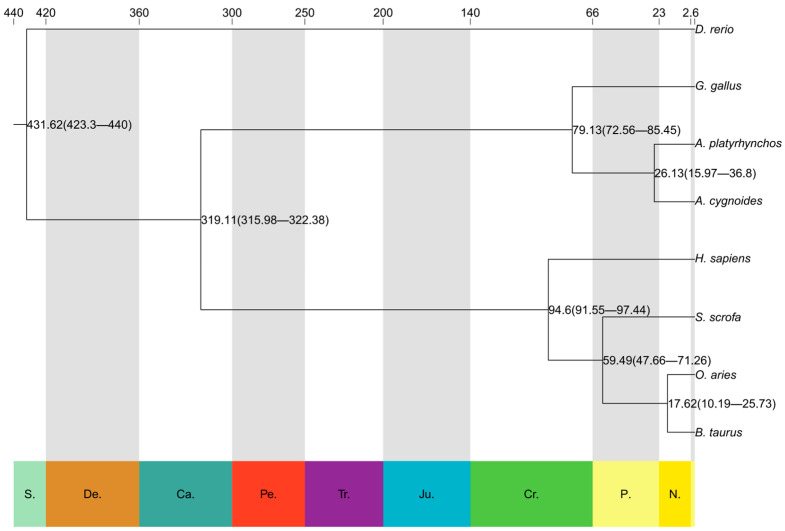
Phylogenetic trees illustrate estimated species divergence times. The numbers at the top of the figure represent estimated divergence times (in millions of years ago, Mya), with larger values indicating earlier divergence events. The branches depict the evolutionary relationships among species, and the values along the branches represent the divergence time ranges between species, with specific intervals shown in parentheses. Abbreviations of geological periods are displayed at the bottom of the figure, including N. (Neoproterozoic), Ca. (Cambrian), S. (Silurian), De. (Devonian), Tr. (Triassic), Ju. (Jurassic), Cr. (Cretaceous), and P. (Paleoproterozoic).

The evolutionary tree of divergence time was constructed using PAML software (v4.9i) (Figure 4), and the results showed that zebrafish diverged the earliest as an outgroup species, with a divergence time of about 431.62 Mya (millions of years ago); poultry and mammals diverged at a time of about 319.11 Mya; humans diverged at a time of about 94.6 Mya; chicken diverged at a time of about 79.13 Mya; pig diverged at a time of about 59.49 Mya; for duck and geese, it was about 26.13 Mya; and for cow and sheep, it was about 17.62 Mya.

### 3.5. Genome-Wide Duplication Events Between Chickens and Other Species Analyzed Based on the Ks Approach

**Figure 5:**
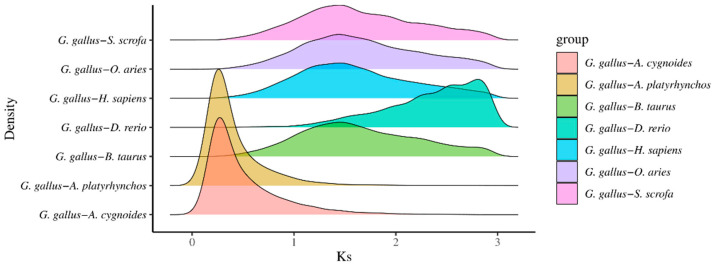
Distribution of Ks values among chicken and other species. The x-axis (Ks) represents the synonymous substitution rate, while the y-axis (density) indicates the distribution density. Colored density curves correspond to the Ks distributions between chicken and each of the other species analyzed. By examining the peak positions of the Ks distributions, the timing of whole-genome duplication events and the evolutionary relationships between species can be inferred.

Whole-genome duplication (WGD) events between chickens and seven other species—goose, duck, cow, zebrafish, human, sheep, and pig—were analyzed using the synonymous substitution rate (Ks) method. The estimated timing of these WGD events was calculated using the formula T = Ks/2r, where r represents the neutral substitution rate. The results are presented in Figure 5. Based on the Ks distribution curves, the Ks peak between chickens and both ducks and geese was 0.264, corresponding to an estimated divergence time of approximately 83.02 million years ago (Mya). For chickens and mammals (humans, pigs, sheep, and cows), the Ks peak was 1.44, indicating a divergence time of about 322.40 Mya. The Ks peak between chickens and zebrafish was 2.77, with a corresponding divergence time of approximately 451.33 Mya. These findings suggest that chickens share the most recent common ancestor with ducks and geese, are more distantly related to mammals, and are most distantly related to zebrafish. This pattern is consistent with the evolutionary relationships inferred from the phylogenetic tree analysis.

### 3.6. Comparison of Genomic Structural Differences Between Chickens, Ducks, and Geese Based on Genomic Collinearity Analysis

**Figure 6:**
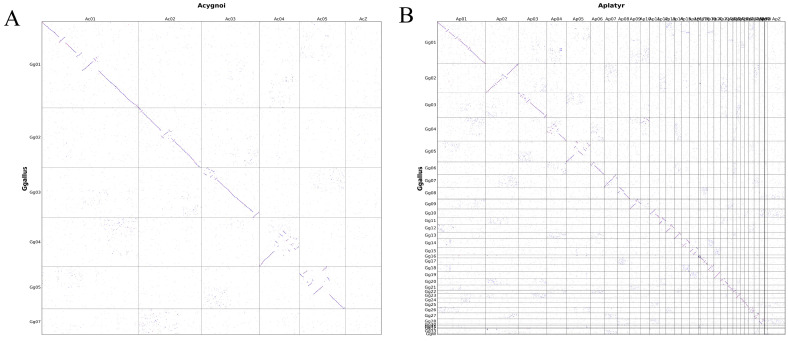
Genomic collinearity is plotted between chickens and closely related species. (**A**) Collinearity between chickens and geese. (**B**) Collinearity between chickens and ducks. The horizontal and vertical axes represent chromosomal positions in each species. Each dot indicates a pair of homologous genes exhibiting collinearity between the two genomes.

A genomic collinearity analysis between chickens and both geese and ducks revealed notable structural similarities and differences. In the comparison between chickens and geese, strong collinearity was observed between chromosomes 1 and 2, indicating high genomic conservation in these regions. In contrast, fewer collinear blocks were detected on chromosomes 4 and 7, suggesting weaker collinearity, potentially due to structural variations such as chromosomal recombination, deletion, or translocation (Figure 6A). This finding provided important clues for the evolution of different traits in chickens and geese. Similarly, in the comparison between chickens and ducks, densely distributed collinear regions were found on chromosomes 1, 2, and 5, indicating strong genomic conservation in these areas. However, chromosomes 16 and 19 showed markedly fewer syntenic regions, suggesting weaker collinearity and possible chromosomal structural variations (Figure 6B), which provided important clues for the formation of different traits between chickens and ducks during the evolutionary process.

### 3.7. Distribution of LTR-RT Insertion Ages in the Chicken Genome

**Figure 7:**
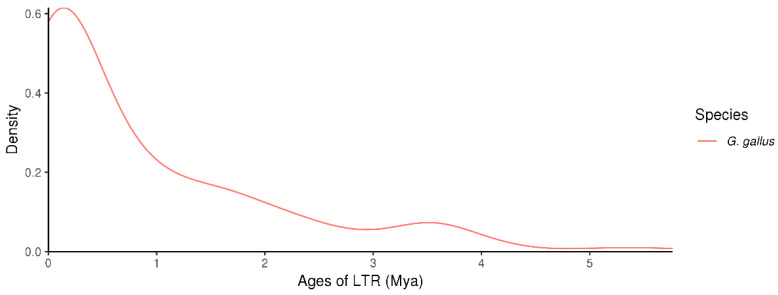
Distribution of LTR insertion times in the chicken genome. This figure illustrates the estimated insertion times and distribution density of LTR retrotransposons in Gallus gallus (chicken). The x-axis represents the insertion time in millions of years ago (Mya), while the y-axis indicates the density of LTR insertions across the genome.

Candidate long terminal repeat retrotransposon (LTR-RT) sequences were identified in the chicken genome to obtain high-confidence LTR-RTs and estimate their insertion times. The results are presented in Figure 7. The distribution of the LTR-RT insertion ages was predominantly concentrated within the recent evolutionary period (i.e., less than 1 million years ago), indicating that LTR-RTs have been highly active in the chicken genome during recent evolutionary history. This pattern suggests that recent LTR-RT insertions may have played an important role in shaping the genome structure and function of chickens. In contrast, the frequency of insertions during the earlier periods (approximately 2–5 million years ago) was significantly lower. This decline may be attributed to the accumulation of mutations or deletions over time, which can lead to the inactivation or degradation of ancient LTR-RTs, making them more difficult to detect in current genomic analyses.

### 3.8. Association Analysis Between Candidate Genes and Important Chicken Traits

#### 3.8.1. Functional Annotation of Candidate Genes

Based on the KOG, GO, and KEGG databases, we functionally annotated the chicken-specific gene family members, the significantly expanded gene families during evolution, along with the genes identified as being under positive selection. The goal was to explore their potential molecular functions, biological processes, and metabolic pathways, and to further elucidate their associations with key traits in chicken.

**Figure 8:**
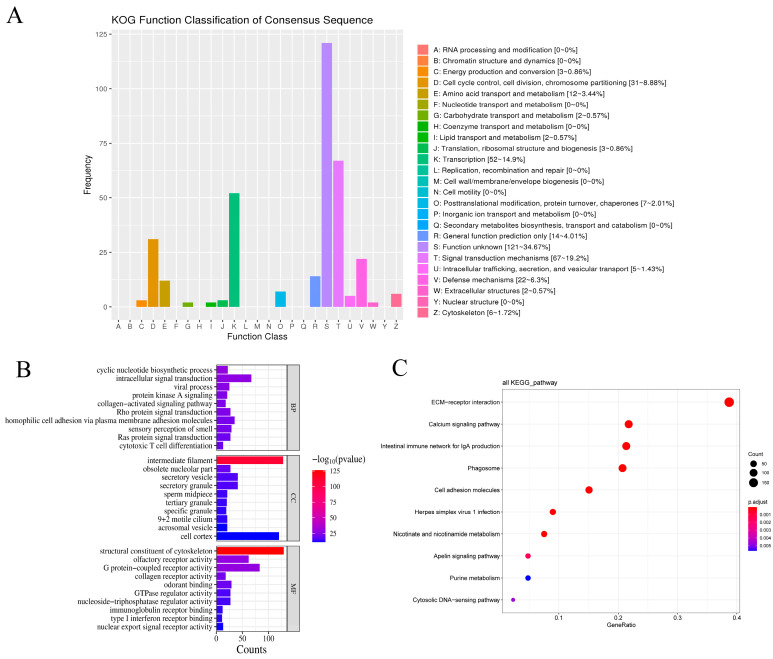
Functional annotation and enrichment analysis of candidate genes. (**A**) KOG functional classification of candidate genes. (**B**) Gene Ontology (GO) functional enrichment analysis. (**C**) KEGG pathway enrichment analysis.

KOG functional classification revealed that these genes were primarily enriched in categories such as Transcription, Signal Transduction Mechanisms, Cell Cycle Control, Cell Division, and Chromosome Partitioning (Figure 8A).

The GO enrichment analysis showed that the significantly enriched biological processes included cyclic nucleotide biosynthetic processes, intracellular signal transduction, viral processes, and the protein kinase A signaling pathway (Figure 8B; Supplementary Table S3). The significantly enriched cellular components were mainly associated with intermediate filaments, secretory vesicles, secretory granules, and the sperm midpiece (Figure 8B; Supplementary Table S4). In terms of molecular functions, enrichment was observed in structural constituents of the cytoskeleton, olfactory receptor activity, G protein-coupled receptor activity, collagen receptor activity, and odorant binding (Figure 8B; Supplementary Table S5)

The KEGG pathway enrichment analysis indicated that the candidate genes were predominantly involved in pathways such as the ECM–receptor interaction, the intestinal immune network for IgA production, phagosome–lysosome fusion, and the calcium signaling pathway (Figure 8C; Supplementary Table S6).

#### 3.8.2. Combining Functional Annotation and a Literature Search to Explore Candidate Gene–Trait Associations

In this study, a number of candidate genes associated with important traits in chickens, and which may play essential roles in the development of diverse phenotypic traits, were identified through various comparative genomics approaches. Based on functional annotations from the KOG, GO, and KEGG databases, combined with a comprehensive literature review, the relationships between these candidate genes and key traits were further clarified.

The results showed (Supplementary Table S7) that 27 genes, including *TBX22*, *ADCY10L1*, *LCORL*, *AGBL4*, *AGBL5*, *CCK*, and *COL1A1*, were mainly involved in biological processes such as the cyclic nucleotide biosynthesis process and intracellular signaling, and were involved in important pathways such as the calcium signaling pathway and metabolism. Through the functional annotation analysis 34 genes, including *A-FABP*, *H-FABP*, *FK1L*, *AMPD1*, *PRKAB2*, *LPL*, *BF1*, and *MHCIA2*, were found to be mainly involved in biological processes, such as protein kinase A signaling and Rho protein signaling, and signaling pathways, such as cytoskeletal structure and ECM–receptor interactions. Twenty-one genes, including *IGF-1*, *FFAR2L*, *GPR42L*, *HEATR7B1*, *MROH2BL23*, and *WDR25*, are mainly located in cellular components such as spermatid, type 9+2 motile cilia, and acrosomal vesicles, and are involved in signaling pathways such as G protein-coupled receptor activity. Another 55 genes, including *C1QBP*, *PCBD2*, *TCD8AL1*, *TCRA*, *CHIR-B2*, *TCD8AL5*, *DUSP1*, and *C14H16orf62*, are mainly enriched in cellular components such as secretory vesicles, secretory granules, tertiary granules, and specific granules, and are involved in cytotoxic T cell differentiation, type I interferon receptor binding, immunoglobulin binding, and biological processes involving signaling pathways such as herpes simplex virus type 1 infection and phagosomes.

## 4. Discussion

In this study, the candidate genes associated with important traits in chickens were identified through comparative genomic analyses, including gene family clustering, gene family expansion and contraction, and a positive selection analysis. Functional annotation using the KOG, GO, and KEGG databases further clarified the potential relationships between these candidate genes and key economic traits in chickens.

Growth traits are among the core economic traits in chicken breeding [35], as they can significantly improve feed conversion efficiency and effectively reduce production costs through precise genetic selection [36]. In this study, 27 candidate genes were identified as potentially associated with growth traits, including *TBX22* and *ADCY10L1*, which are primarily involved in cyclic nucleotide biosynthesis and intracellular signaling pathways, such as calcium signaling and metabolic regulation. Previous studies have shown that cyclic nucleotide biosynthesis plays a central role in the production of intracellular second messengers, including cyclic adenosine monophosphate (cAMP) and cyclic guanosine monophosphate (cGMP) [37]. These molecules are key regulators of various physiological functions and exert their effects by activating protein kinase A (PKA) and protein kinase G (PKG), which in turn influence muscle cell proliferation, differentiation, and energy metabolism [37]. Among the candidate genes identified, 13, including *TBX22* [38], *LCORL* [38], *AGBL4* [39], and *GH* [40], have previously been reported in association with chicken growth traits, further supporting the relevance of our findings. For example, Cao et al. [38] conducted a genome-wide association study (GWAS) using resequencing data and 8-week body weight records from 900 Goodyear hens. They identified eight key candidate genes—*TBX22*, *FGFBP1*, *FGFBP2*, *HTR2C*, *APBB2*, *LCORL*, *HMX1*, and *NCAPG*—that are associated with early growth traits in Goodyear chickens. Similarly, Tan et al. [41] performed a GWAS using a high-density 600K SNP microarray in two local breeds, Ninghai Yellow Chickens and Guangxi Yellow Chickens, applying a general linear model to assess associations with growth traits. They identified four candidate genes—*ADM*, *AGBL4*, *ASCC3*, and *WTIP*—as being significantly related to growth performance in these breeds.

Meat quality in chickens—including traits such as tenderness, texture, and flavor—is a key factor influencing industrial competitiveness [42]. In this study, 34 candidate genes related to meat quality were identified, including *A-FABP* and *H-FABP2*. These genes are primarily involved in biological processes such as protein kinase A signaling and Rho protein signaling and are enriched in the pathways related to cytoskeletal organization and ECM–receptor interactions. Previous studies have shown that the extracellular matrix (ECM), mainly composed of collagen, fibronectin, and laminin, plays a critical role in maintaining muscle tissue integrity and mechanical properties, directly influencing tenderness and toughness [43]. The integrin family, as primary ECM receptors, regulates muscle fiber growth and alignment through downstream signaling molecules such as focal adhesion kinase (FAK) and Rho GTPases. Moreover, the collagen content and degree of cross-linking in the ECM are key determinants of muscle shear force [43]. The cytoskeleton—comprising actin filaments, microtubules, and intermediate filaments—also plays a crucial role in muscle fiber contractility and postmortem muscle stiffness [44]. Among the identified candidate genes, seven—including *A-FABP* [45], *H-FABP* [45], *PRKAB2* [46], *CAPN1* [47], *ANXA6* [48], *ACAA2* [47], and *LPL* [47]—have previously been reported to be associated with chicken meat quality, supporting the relevance of our findings. The *H-FABP* gene is located on chromosome 23 in chickens and comprises four exons encoding a total of 133 amino acids [48]. It is primarily expressed in cardiac and skeletal muscle tissues, where it binds fatty acids and plays a key role in fatty acid metabolism. Ye et al. [45] investigated the relationship between single nucleotide polymorphisms (SNPs) in the *A-FABP* and *H-FABP* genes and intramuscular fat (IMF) content in Pekinese chickens. They found that the *A-FABP* BB genotype and the *H-FABP* CD genotype were significantly associated with increased IMF content, suggesting a potential interaction between the two genes in regulating fat deposition, although further research is needed to clarify their combined effects. The *PRKAB2* gene belongs to the protein kinase family and is involved in key physiological processes such as energy regulation, fatty acid oxidation, and glucose metabolism. It has been recognized as an important determinant of meat quality traits in livestock [49]. Wang et al. [46] used sequencing and single-strand conformation polymorphism (SSCP) analysis to examine genetic variation in the *PRKAB2* gene across five purebred lines and three crossbred lines of Daheng high-quality broilers. Their results revealed a conserved coding sequence (CDS) region and identified a T-C point mutation at position 406 bp in exon 1. The wild-type allele (T) at this locus was found to be associated with improved meat tenderness, highlighting the gene’s potential utility in marker-assisted selection for meat quality.

Reproductive traits are among the most important economic traits in chickens, directly influencing key indicators such as reproductive efficiency, hatchability, and egg production. Reproductive performance not only determines overall productivity but also has a significant impact on the economic returns of poultry farming [50]. In this study, 21 candidate genes potentially related to reproductive traits were identified, including *IGF-1*, *FFAR2L*, and *GPR42L*. These genes were primarily localized in cellular components such as the sperm midpiece, 9+2-type motile cilia, and acrosomal vesicles, and were enriched in the signaling pathways involving G protein-coupled receptor (GPCR) activity. GPCRs are critical signaling molecules in sperm cells, and their activation promotes calcium ion (Ca²⁺) influx and enhances sperm motility via the cAMP-KA signaling pathway [51]. Additionally, the binding of glycoproteins to GPCRs on the sperm surface can activate the PLC-P3-AG-KC signaling cascade, which facilitates the release of hydrolytic enzymes from acrosomal vesicles, enabling sperm to penetrate the egg membrane [52]. The sperm midpiece, which is rich in mitochondria, provides ATP to support sperm motility. Activation of the glucagon-like peptide-1 receptor (GLP-1R) has been shown to enhance mitochondrial oxidative phosphorylation, thereby increasing sperm motility [52]. Acrosomal vesicles, lysosome-like organelles located in the sperm head, play a key role in egg penetration by releasing enzymes that degrade the zona pellucida. Among the candidate genes identified, five, including *IGF-1* [53], *SLC25A29* [54], *WDR25* [55], *CAMK2D* [56], and *ENC1* [56], have been previously reported in association with chicken reproductive traits, providing further evidence for their potential functional relevance. For example, Cao et al. [57] conducted a genome-wide association study (GWAS) of chicken reproductive traits using a 600K SNP microarray and identified two candidate genes, *T8SIA1* and *ENC1*, associated with reproductive performance. Similarly, Wu et al. [53] used PCR–RFLP technology to detect single nucleotide polymorphisms in the *GNRHR* and *IGF-1* genes in Wenchang chickens. Their study revealed that *GNRHR* significantly influenced egg production at both 300 and 400 days of age, while *IGF-1* was significantly associated with egg production at 300 days of age.

Disease resistance is a key factor influencing health, productivity, and the overall performance of chickens [58]. Common infectious diseases such as Newcastle disease, avian influenza, and fowl dysentery not only reduce economic returns in poultry production but also pose threats to animal welfare and public health [59]. In this study, 55 candidate genes associated with disease resistance were identified, including *C1QBP*, *CHIR-B2*, and *PCBD2*. These genes were primarily annotated to cellular components such as secretory vesicles, secretory granules, tertiary granules, and specific granules. They were involved in biological processes such as cytotoxic T cell differentiation, type I interferon receptor binding, and immunoglobulin binding, and enriched in immune-related pathways including herpes simplex virus type 1 infection and phagolysosome formation. For example, a herpes simplex virus type 1 infection triggers the upregulation of interferon-stimulated genes and inhibits viral replication through the binding of viral proteins to host receptors (e.g., nectin-1 or HVEM) and a subsequent activation of the JAK-STAT signaling pathway [60]. Cytotoxic T lymphocytes (CTLs) are crucial in antiviral defense. Upon infection, viral peptides (such as HSV-1 g B protein) are presented by MHC class I molecules, leading to the activation and differentiation of CD8⁺ T cells into CTLs [61]. Among the candidate genes, 12—including *C1QBP* [62], *VAV2* [63], *IL12B* [63], *DUSP1* [63], *ZBED1* [64], *MX1* [64], *GRAP2* [64], *TEDC1* [64], *RASSF5* [64], *IFT22* [64], *ZFHX4* [64], and *ALB* [64]—have been previously reported in association with disease resistance traits in chickens, supporting their potential functional roles in immune regulation and pathogen defense. Heterophilic cells (H/L ratio) are avian-specific immune cells that play a crucial role in pathogen phagocytosis and immune defense. Wang et al. [62] conducted a GWAS on 1,317 Kobo broilers using a 55K SNP array and identified *C1QBP* as a candidate gene involved in H/L regulation. Newcastle disease is a highly contagious viral infection in poultry, and advances in molecular quantitative genetics, offer promising strategies for breeding disease-resistant chickens. Walugembe et al. [63] performed a GWAS on 1,440 indigenous Ghanaian chickens and identified 12 quantitative trait loci (QTL) associated with the suppression of a Newcastle disease virus (NDV) response. Through gene annotation, five key candidate genes—*HORDC1*, *VAV2*, *IL12B*, *DUSP1*, and *IL17B*—were found to be associated with immune resistance to NDV. Similarly, Habimana et al. [64] conducted a GWAS on 185 Rwandan local chickens and discovered that NDV antibody production was associated with the genomic regions on chromosome 1. Additionally, 10 NDV-associated SNPs were identified across chromosomes 2, 4, 8, 13, 17, 19, and 26. A gene annotation of these loci revealed 14 candidate genes—*CDC16*, *ZBED*1, *MX1*, *GRAP2*, *UBAC1*, *TEDC1*, *RASSF5*, *IFT22*, *JARID2*, *GABRB2*, *ADCYAP1*, *PCBD2*, *ZFHX4*, and *ALB*—that may contribute to antibody production and immune response to NDV in Rwandan chickens.

Although this study identified a number of candidate genes associated with growth, meat quality, reproductive performance, and disease resistance in chickens, it is important to consider the potential negative effects of the over-selection of certain genes. For example, *TBX22* was found to be associated with reproductive traits and *C1QBP* with disease resistance in chickens; however, these genes have different biological functions in other species. For example, *TBX22* is associated with facial developmental defects such as a cleft lip and a cleft palate in humans [65], whereas *C1QBP* plays an essential role in regulating mitochondrial morphology, metabolism, and autophagy [66]. Although the above functions have not been observed in chickens, it should be recognized that the function of the same gene may differ in different species. In addition, a balance of trait selection should be maintained in genetic selection to avoid excessive concentration on a single trait. For example, the over-pursuit of improvement in growth traits may lead to reduced fertility, health problems, and even animal welfare issues in the species [67]. To ensure the sustainability of poultry breeding programs, breeders should adopt more integrated and prudent strategies to reduce potential risks.

## 5. Conclusions

In this study, using high-quality genomic data from several species, we screened several candidate genes related to important traits in chickens, such as *TBX22*, *ADCY10L1*, *LCORL* and *AGBL4*, by using comparative genomics methods. Through annotation and finding the functions of candidate genes, we concluded the following: genes such as *TBX22*, *LCORL*, and *GH* are related to growth traits in chickens; *A-FABP*, *H-FABP*, *PRKAB2*, and other genes are related to chicken meat quality; *IGF-1*, *SLC25A29*, *WDR25*, and other genes are related to chicken reproductive traits; and *C1QBP*, *VAV2*, *IL12B*, and other genes are related to chicken disease resistance traits. These findings provide valuable genetic resources and theoretical support for future studies on trait improvement and molecular breeding in chickens.

## Figures and Tables

**Table 1:** Statistics of gene family clustering results.

Item	*A. cygnoides*	*A. platyrhynchos*	*B. taurus*	*D. rerio*	*G. gallus*	*H. sapiens*	*O. aries*	*S. scrofa*
Number of genes	18,047	16,739	21,535	24,154	17,489	23,772	21,208	20,447
Number of genes in orthogroups	17,125	15,899	20,334	19,263	16,050	22,271	20,182	19,079
Number of unassigned genes	922	840	1201	4891	1439	1501	1026	1368
Percentage of genes in orthogroups	94.90	95.00	94.40	79.80	91.80	93.70	95.20	93.30
Percentage of Unassigned genes	5.10	5.00	5.60	20.20	8.20	6.30	4.80	6.70
Number of orthogroups containing species	14,520	13,796	16,888	12,352	13,742	16,397	16,747	16,169
Percentage of othogroups containing species	65.40	62.10	76.00	55.60	61.90	73.80	75.40	72.80
Number of species- specific orthogroups	148	71	68	751	127	275	35	82
Number of genes in species-specific orthogroups	741	258	314	4017	824	1563	179	432
Percentage of genes in species-specific orthogroups	4.10	1.50	1.50	16.60	4.70	6.60	0.8	2.10

**Table 2:** Selected results of positively selected genes in chickens during the evolutionary process.

Gene	Branch *p*-Value	Locus Information	Gene Family
*GTSF1*	0.00065	8, L, 0.995 ** 46, E, 0.965 * 83, Q, 0.992 **	OG0002511
*GRAP2*	0.0034	20, S, 0.983 * 21, G, 0.990 **	OG0004707
*RCN1L*	0.036	16, L, 0.952 *	OG0004041
*AGBL4*	0.0454	64, G, 0.963 *	OG0005294
*ACBD6*	0.0016	11, Y, 0.985 * 589, R, 0.969 * 590, K, 0.990 **	OG0003321
*RP11-6L6.2*	0.0013	145, Q, 0.974 *	OG0004058
*MYRF*	0.00017	16, Q, 0.961 * 31, L, 0.976 * 36, A, 0.971 *	OG0004190
*GCFC2*	0.00002	14, P, 0.960 * 19, G, 0.957 * 69, Q, 0.983 *	OG0004841
*CFAP410*	0.045	410, E, 0.960 * 411, L, 0.962 *	OG0005392

Note: * represents significance greater than 0.95, ** represents highly significant and significance greater than 0.99.

## Data Availability

All data are included in this paper.

## Supplementary Materials

The following supporting information can be downloaded at https://www.mdpi.com/article/10.3390/genes16060627/s1: Supplementary Table S1: Species details statistics; Supplementary Table S2: Genome Download Links; Supplementary Table S3: Statistics of GO biological process details; Supplementary Table S4: Detailed statistics of GO cell components; Supplementary Table S5: Statistics of GO molecule function details; Supplementary Table S6: Statistics of KEGG signaling pathway details; and Supplementary Table S7: Statistics of candidate genes related to important traits in chickens.
