# Supplementary material for: Comparative Genomic Analysis Across Multiple Species to Identify Candidate Genes Associated with Important Traits in Chickens
Source: Genes (Basel). 2025 May 24;16(6):627. doi: 10.3390/genes16060627 (PMC12192435; doi:10.3390/genes16060627)
Supplement: Supplementary file 1 [file genes-16-00627-s001.zip › Supplementary tables.pdf]

Supplementary Table S1 Species details statistics
Supplementary Table S2 Genome Download Links
Supplementary Table S3 Statistics of GO biological process details
Supplementary Table S4 Detailed statistics of GO cell components
Supplementary Table S5 Statistics of GO molecule function details
Supplementary Table S6 Statistics of KEGG signaling pathway details
Supplementary Table S7 Statistics of candidate genes related to important traits in chicken
